# Frajunolides L–O, Four New 8-Hydroxybriarane Diterpenoids from the Gorgonian *Junceella fragilis*

**DOI:** 10.3390/md9091477

**Published:** 2011-09-02

**Authors:** Chia-Ching Liaw, Yao-Haur Kuo, Yun-Sheng Lin, Tsong-Long Hwang, Ya-Ching Shen

**Affiliations:** 1 School of Pharmacy, College of Medicine, National Taiwan University, Taipei 100, Taiwan; E-Mail: biogodas@hotmail.com; 2 Department of Marine Biotechnology and Resources, National Sun Yat-Sen University, Kaohsiung 804, Taiwan; E-Mail: x00010106@meiho.edu.tw; 3 National Research Institute of Chinese Medicine, Taipei 112, Taiwan; E-Mail: kuoyh@nricm.edu.tw; 4 Graduate Institute of Natural Products, Chang Gung University, Taoyuan 333, Taiwan; E-Mail: htl@mail.cgu.edu.tw

**Keywords:** *Junceella fragilis*, 8-hydroxybriarane, frajunolides, anti-inflammatory activities

## Abstract

Four new 8-hydroxybriarane diterpenoids, frajunolides L–O (**1**–**4**), were isolated from the Taiwanese gorgonian *Junceella fragilis*. The structures of compounds **1**–**4** were elucidated based on spectroscopic analysis, especially 2D NMR (^1^H-^1^H COSY, HSQC, HMBC and NOESY) and HRMS. Compounds **1** and **4** showed weak anti-inflammatory activity as tested by superoxide anion generation and elastase release by human neutrophil in response to fMLP/CB. Compound **3** showed selective inhibition on elastase release *in vitro*.

## 1. Introduction

A number of secondary metabolites with potential pharmacological activities such as cytotoxic, antiviral, anti-inflammatory, and insecticidal effects were discovered from marine organisms [1]. Marine diterpenoids of the class briarane have been investigated with great interest owing to their novel structures and interesting bioactivities [2–5]. The gorgonian of the genus *Junceella* grown in the tropical and subtropical waters of Indo-West Pacific regions are well known as a source of highly oxidized briarane-type diterpenoids with a γ-lactone moiety [6–9]. In continuation of our study on the chemistry and biological activities of briarane diterpenoids [10–16], we investigated the Taiwanese gorgonian *J. fragilis*. A chemical investigation of the acetone extract has yielded four new 8-hydroxybriarane diterpenoids, designated as frajunolides L–O (**1**–**4**). In this paper, we report the isolation, structural elucidation, and anti-inflammatory activity as tested by superoxide anion generation and elastase release by human neutrophil in response to fMLP/CB, of these compounds.

## 2. Results and Discussion

**Figure 1:**
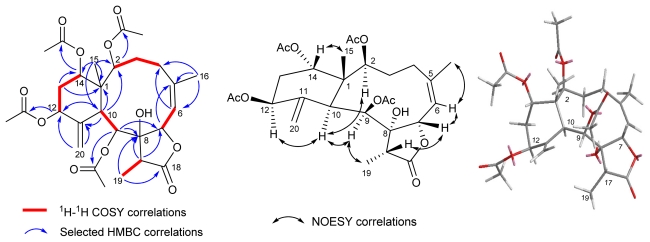
^1^H-^1^H COSY and HMBC correlations of **1**; NOESY correlations and computer-generated perspective model of **1** using MM2 force field calculation.

Compound **1** was deduced to have the molecular formula C_28_H_38_O_11_ with ten degrees of unsaturation from high-resolution ESI mass spectrometry. The IR absorptions were observed at 3439, 1768 and 1735 cm^−1^ suggesting the presence of hydroxyl, γ-lactone and ester groups, respectively. The ^1^H-, ^13^C-NMR and DEPT spectroscopic data (Table 1) revealed that compound **1** possessed four acetyl groups (*δ*_H_ 1.94, 1.98, 2.13, and 2.21), two tertiary methyl protons (*δ*_H_ 1.15, Me-15; *δ*_H_ 2.03, Me-16), a doublet methyl (*δ*_H_ 1.15, *d*, *J* = 6.9 Hz, Me-19), five oxygenated methine protons (*δ*_H_ 4.94, *t*, *J* = 3.3 Hz, H-2; *δ*_H_ 5.28, *d*, *J* = 9.6 Hz, H-7; *δ*_H_ 5.62, *d*, *J* = 5.1 Hz, H-9; *δ*_H_ 5.32, *m*, H-12; 4.77, br *s*, H-14), a trisubstituted olefinic group (*δ*_H_ 5.58, *d*, *J* = 9.6 Hz, H-6; *δ*_C_ 120.0, C-6; *δ*_C_ 145.2, C-5), an oxygenated quaternary carbon (*δ*_C_ 82.6, C-8), an exocyclic double bond (*δ*_H_ 5.34, 5.30, H_2_-20; *δ*_C_ 118.3, C-20; 146.3, C-11), two methine carbons (*δ*_C_ 40.6, C-10; *δ*_C_ 43.2, C-17), three methylene carbons (*δ*_C_ 30.8, C-3; *δ*_C_ 29.0, C-4; *δ*_C_ 33.5, C-13), along with a γ-lactone carbonyl carbon (*δ*_C_ 175.9, C-18). The proton and carbon assignments of **1** were completely established by using 1D- and 2D NMR experiments, including ^1^H-^1^H COSY, HSQC, and HMBC (Figure 1). The ^1^H-^1^H COSY spectrum exhibited four sets of correlations (H-2/H-3/H-4, H-6/H-7, H-9/H-10, and H-12/H-13/H-14). The HMBC correlations of Me-15 (*δ*_H_ 1.15, *s*)/C-1 (*δ*_C_ 47.0), C-2 (*δ*_C_ 74.2), C-10 (*δ*_C_ 40.6), C-14 (*δ*_C_ 73.6); Me-16 (*δ*_H_ 2.03, *s*)/C-4 (*δ*_C_ 29.0), C-5 (*δ*_C_ 145.2), C-6 (*δ*_C_ 120.0); Me-19 (*δ*_H_ 1.15, *d*, *J* = 6.9 Hz)/C-8 (*δ*_C_ 82.6), C-18 (*δ*_C_ 175.9); H-9 (*δ*_H_ 5.62, *d*, *J* = 5.1 Hz)/C-8, C-7 (*δ*_C_ 78.0); H-10 (*δ*_H_ 3.57, *d*, *J* = 5.1 Hz)/C-1, C-2, C-11 (*δ*_C_ 146.3), C-12 (*δ*_C_ 71.5); H_2_-20 (*δ*_H_ 5.34, s; 5.30, s)/C-11, C-12; H-13 (*δ*_H_ 1.95, *m*; 2.20, *m*)/C-14, C-1 established the connectivities from C-1 to C-20 unambiguously, and revealed that compound **1** belongs to 8-hydroxybriarane diterpenoids with a γ-lactone ring [11]. The four acetate groups of **1** were assigned at C-2, C-9, C-12, and C-14 positions by the HMBC correlations between the acetate carbonyl carbons (*δ*_C_ 170.4 × 2, 170.3, and 168.9) and four oxygenated methine protons (*δ*_H_ 4.94, H-2; *δ*_H_ 5.62, H-9; *δ*_H_ 5.32, H-12; 4.77, H-14). Thus the planar structure of **1** was completely established.

Our results showed that the planar structure of compound **1** is the same as frajunolide A, but differing in the ^1^H- and ^13^C NMR data of the methylenecyclohexane ring, especially at C-12 and C-20 positions [10]. The ^13^C NMR chemical shift of C-12 (*δ*_C_ 71.5) was shifted downfield in comparison with frajunolide A (*δ*_C_ 67.3), suggesting that the relative stereochemistry of H-12 was *α*-orientation [11]. The relative configuration of **1** was determined by NOESY correlations (Figure 1) and MM2 minimized energy calculated molecular modeling, and comparison with other naturally occurring briarane diterpenoids [2–5]. Briarane-type diterpenoids were reported to have the Me-15 in the *β*-orientation and H-10 in the *α*-orientation. In the NOESY of **1**, H-10 showed correlations with H-2, H-9, H-12, suggesting that these protons are located on the *α*-face. In addition, the correlation between H-9 and Me-19 indicated that Me-19 is *α*-oriented too. However, correlation of H-17/H-7 suggested that H-7 and H-17 are on the *β*-face. Moreover, NOESY correlation of H-14/Me-15 suggested that 14-acetoxy group is located on the *α*-face. The *Z*-configuration at C-5 was elucidated by the observation of NOESY correlation between H-6 and Me-16. On the basis of the above interpretation, the structure of compound **1** was elucidated. The name frajunolide L was given.

**Figure 2:**
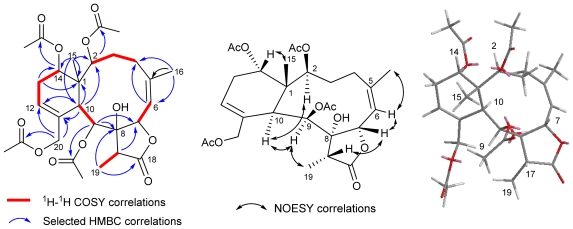
^1^H-^1^H COSY and HMBC correlations of **2**; NOESY correlations and computer-generated perspective model of **2** using MM2 force field calculation.

Compound **2** had the molecular formula C_28_H_38_O_11_, the same as that of **1**, as determined by HRESIMS, suggesting that the structure of **2** was similar to **1**. The IR spectrum of **2** also displayed strong absorptions at 3429, 1776 and 1735 cm^−1^ indicating that compound **2** contained hydroxyl and carbonyl groups of five-membered γ-lactone ring and ester groups. Both ^1^H- and ^13^C NMR spectroscopic data (Table 1) of **2** were found to be similar to those of **1**. These signals include four acetyl group (*δ*_H_ 1.92, 2.01, 2.07, and 2.16), two tertiary methyl protons (*δ*_H_ 0.99, Me-15; *δ*_H_ 1.99, Me-16), a methyl doublet (*δ*_H_ 1.17, *d*, *J* = 6.8 Hz, Me-19), and a methine quartet (*δ*_H_ 1.15, *q*, *J* = 7.2 Hz). However, 1D- and 2D-spectroscopic data of **2** revealed that the exocyclic double bond (C-11/C-20) in **1** shifted to C-12 (*δ*_C_ 127.6)/C-11 (*δ*_C_ 134.2) and an acetate group was found to locate at C-20 (*δ*_C_ 68.7). The gross structure of **2** was further deduced from the ^1^H-^1^H COSY, HMQC, HMBC correlations (Figure 2). The relative configuration of **2** was determined by NOESY correlations (Figure 2) and application of MM2 molecular modeling together with comparing the NMR spectra of **2** with those of **1**. The NOESY correlations of H-10/H-2, H-9, and H-9/Me-19 suggested that the configurations of H-2, H-9, H-10, and Me-19 were in *α*-orientation while correlations of H-7/H-6, H-17, and H-14/Me-15 agreed with *β*-disposition of H-7, H-14, Me-15 and H-17.

**Figure 3:**
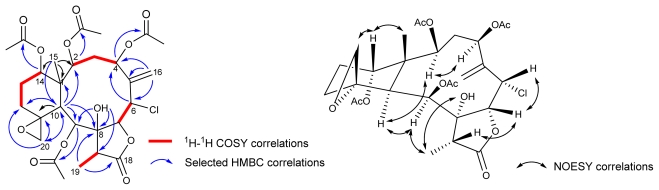
^1^H-^1^H COSY, HMBC, and NOESY correlations of **3**.

The HRESI mass spectrum of **3** gave a *quasi*-molecular ion peak at *m/z* 589.2266 [M + Na]^+^, indicative of a molecular formula C_28_H_38_ClO_12_ (calc. for *m/z* 589.2261), consistent with 10 degrees of unsaturation. The presence of a chloride was evident from the fragment [M + Na]^+^ at *m/z* 589 and the isotope fragment [M + Na + 2]^+^ at *m/z* 591 in ESIMS, with the typical ratio of relative intensity (3:1) in the mass spectrum. In the infrared spectrum, strong absorption bands were found at 3436, 1735 and 1780 cm^−1^ characteristic for hydroxyl, ester carbonyl (acetyl) and five-membered γ-lactone ring, suggesting a briarane-type diterpenoid similar to compounds **1** and **2**. It was found that the ^1^H- and ^13^C NMR spectra of **3** in CDCl_3_ showed mostly broad peaks and in some cases, certain peaks were not observed. In order to mark more optimum signals of the NMR spectra, compound **3** was dissolved in pyridine-*d*_5_. The ^1^H- and ^13^C NMR data (Table 1) of **3** revealed the presence of four acetate groups (*δ*_H_ 1.99, 2.11, and 2.30 × 2; *δ*_C_ 172.5, 171.1 × 2, 170.3, 22.2, 21.9, 21.8, and 21.5), an exocyclic double bond (*δ*_H_ 5.83, 5.42, H_2_-16; *δ*_C_ 144.0, C-5; 125.5, C-16) and a γ-lactone carbonyl carbon (*δ*_C_ 176.4, C-19). Judging from the molecular formula and NMR data of **3**, six degrees of unsaturation were counted for, indicating that compound **3** contained a tetracyclic system including an exocyclic epoxide (*δ*_H_ 2.84, *d*, *J* = 4.0 Hz; 2.59, br s, H_2_-20; *δ*_C_ 58.2, C-11; 52.6, C-20). The HMBC correlations (Figure 3) between H-2 (*δ*_H_ 6.68, *d*, *J* = 8.5 Hz), H-4 (*δ*_H_ 5.90, *d*, *J* = 10.5 Hz), H-9 (*δ*_H_ 6.30, *s*), and H-14 (*δ*_H_ 5.25, *s*) with one of ester carbonyl carbons, respectively, revealed that four acetyl groups were connected to the C-2, C-4, C-9, and C-14 positions. By interpretation of the NMR spectroscopic data, the planar structure of compound **3** was elucidated. The relative configuration of **3** was determined by NOESY (Figure 3) and detailed comparison with known compounds [10]. The chemical shift of C-11 (*δ*_C_ 58.2) and C-20 (*δ*_C_ 52.6), and the NOESY correlations between H_2_-20 and Me-15 agreed with *β*-face of H_2_-20, 11*R*-configuration regarding the exocyclic epoxide, and chair conformation of the cyclohexane ring. Furthermore, NOESY correlations of H-10/H-2, H-4/H-2 and H-10/H-9 suggested that H-2, H-4 and H-9 were located on the same face and could be assigned as α.

**Figure 4:**
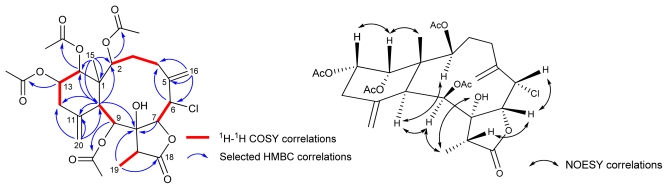
^1^H-^1^H COSY, HMBC, and NOESY correlations of **4**.

Compound **4** showed a pair of *quasi*-molecular ion peaks at *m/z* 607 and 609 [M + H]^+^ with a ratio of 3:1 in the ESIMS, indicating the presence of a chlorine atom. Moreover, a molecular formula C_28_H_37_ClO_11_ was established by HRESIMS and confirmed by ^1^H- and ^13^C NMR spectroscopic analysis (Table 1). The IR absorption bands at 3467, 1780 and 1739 cm^−1^ indicated that **4** contained hydroxyl, γ-lactone, and ester carbonyl functionalities similar to **1**–**3**. Detailed inspection of ^1^H- and ^13^C NMR spectroscopic data revealed the presence of the key structural feature of a 8-hydroxybriarane diterpenoid with two exocyclic double bonds. The locations of the two exocyclic double bonds were confirmed by the HMBC experiment (Figure 4), which showed correlations of H_2_-16 (*δ*_H_ 4.92, *s*; 5.49, *s*)/C-4 (*δ*_C_ 33.4), C-5 (*δ*_C_ 144.9), and C-6 (*δ*_C_ 56.2), and H_2_-20 (*δ*_H_ 4.92, *s*; 5.19, *s*)/C-10 (*δ*_C_ 44.6), C-11 (*δ*_C_ 147.0), and C-12 (*δ*_C_ 38.6), respectively. In addition, the oxygenated methine proton H-2 (*δ*_H_ 6.62, *d*, *J* = 8.0 Hz), H-9 (*δ*_H_ 6.28,), H-13 (*δ*_H_ 5.72, *ddd*, *J* = 12.0, 5.2, 3.2 Hz), and H-14 (*δ*_H_ 5.66, *s*) showed HMBC correlations with the acetate carbonyl carbons (*δ*_C_ 171.8, 170.9, 170.8, 170.3). Furthermore, detailed analysis of 2D NMR spectroscopic data (^1^H-^1^H COSY and HMBC) established the planar structure of **4**. The configuration of compound **4** was determined on the basis of NOESY correlations (Figure 4). The NOESY correlations of Me-15 (*δ*_H_ 1.27, *s*)/H-14 and H-13/H-14 implied that H-13 and H-14 are on the *β*-face while correlations of H-2/H-10, H-10/H-9, H-9/Me-19, H-17/H-7 and H-6/H-7 confirmed that the configuration of these protons are identical to those of compound **3**.

General pharmacological study of the anti-inflammatory activities of compounds **1**–**4** were evaluated by measuring superoxide anion generation and elastase release by human neutrophils in response to fMet-Leu-Phe (fMLP)/Cytochalasin B (CB) [17]. The results are illustrated in Table 2. Compounds **1** and **4** showed mild inhibitory effects on both superoxide anion generation and elastase release at 10 μg/mL. It is notable that compound **3** exhibited selective but modest inhibition of elastase release *in vitro*.

## 3. Experimental Section

### 3.1. General Experimental Procedures

Optical rotations were recorded on a JASCO DIP-1000 polarimeter. IR spectra were measured on a Hitachi T-2001 spectrophotometer. The ^1^H-^13^C NMR, COSY, HMQC, HMBC, and NOESY spectra were recorded on a Bruker AV-400 or a AV-500 spectrometer, using TMS as internal standard. The chemical shifts are given in *δ* (ppm) and coupling constants (*J*) in Hz. HRESIMS were run on a JEOL JMS-HX 110 mass spectrometer. Silica gel 60 (Merck) was utilized for column chromatography, and precoated silica gel plates (Merck, Kieselgel 60 F-254, 1 mm) were used in preparative TLC. Sephadex LH-20 (Amersham Pharmacia Biotech AB, Sweden) was used for separation and purification of compounds. LiChrospher Si 60 (5 μm, 250-10, Merck) and LiChrospher 100 RP-18e (5 μm, 250-10, Merck) were used in NP-HPLC and RP-HPLC (Hitachi), respectively.

### 3.2. Animal Material

The gorgonian *Junceella fragilis* Ridley (Ellisellidae) was collected in Tai-Tong County, Taiwan, by scuba diving at a depth of 15 meters, in February 2006. The fresh gorgonian was immediately frozen after collection and kept at −20 °C until processing. A voucher specimen (WSG-5) was deposited in the School of Pharmacy, College of Medicine, National Taiwan University, Taipei.

### 3.3. Extraction and Isolation

The gorgonian *J. fragilis* (wet, 2.5 kg) was minced and extracted with acetone (3 × 5 L) at room temperature and the acetone extract was concentrated under vacuum. The crude extract (20 g) was partitioned between EtOAc and H_2_O (1:1). The EtOAc-soluble portion (15 g) was subjected to column chromatography using silica gel and eluted with a gradient of *n*-hexane/EtOAc (10:1 to 0:1) to obtain thirteen fractions (Fr.1~13). Fraction 6 (202 mg) was subjected to RP-HPLC using MeOH/H_2_O (60:40) to give **1** (3.9 mg) and **2** (1.8 mg). Fraction 9 (874 mg) was separated on silica gel column and eluted with gradient *n*-hexane/EtOAc to give seven fractions (Fr. 9-1~6). Fr. 9-4 (157 mg) was purified by RP-HPLC, using solvent mixture of MeOH and H_2_O (65:35) to yield **4** (8.2 mg). Fr. 9-6 (211 mg) was separated on RP-HPLC using MeOH/H_2_O (60:40) to furnish **3** (4.5 mg).

Frajunolide L (**1**): colorless amorphous gum; [α]^24^ _D_ +6.0 (*c* 0.2, CH_2_Cl_2_); IR *ν*_max_ 3439, 2922, 2749, 1768, 1735, 1370, 1248, 1221, 1040 cm^−1; 1^H NMR data (400 MHz, CDCl_3_), see Table 1; ^13^C NMR data (100 MHz, CDCl_3_), see Table 2; ESIMS *m/z* 573 [M + Na]^+^; HRESIMS *m/z* 573.2313 [M + Na]^+^ (calcd for C_28_H_38_O_11_Na, 573.2312).

Frajunolide M (**2**): colorless amorphous powder; [α]^24^ _D_ +8.0 (*c* 0.2, CH_2_Cl_2_); IR *ν*_max_ 3447, 2923, 2853, 1773, 1735, 1645, 1375, 1240, 1223, 1041 cm^−1; 1^H NMR data (400 MHz, CDCl_3_), see Table 1; ^13^C NMR data (100 MHz, CDCl_3_), see Table 2; ESIMS *m/z* 573 [M + Na]^+^; HRESIMS *m/z* 573.2315 [M + Na]^+^ (calcd for C_28_H_38_O_11_Na, 573.2312).

Frajunolide N (**3**): colorless amorphous powder; [α]^24^ _D_ +18.0 (*c* 0.1, CH_2_Cl_2_); IR *ν*_max_ 3436, 2933, 1780, 1735, 1376, 1255, 1235, 1212, 1044, 1017 cm^−1; 1^H NMR data (400 MHz, pyridine-*d*_5_), see Table 1; ^13^C NMR data (100 MHz, pyridine-*d*_5_), see Table 2; ESIMS *m/z* 589 [M + Na]^+^, 591 [M + Na + 2]^+^; HRESIMS *m/z* 589.2266 [M + Na]^+^ (calcd for C_28_H_38_ClO_12_Na, 589.2261).

Frajunolide O (**4**): colorless amorphous gum; [α]^24^ _D_ +6.7 (*c* 0.7, CH_2_Cl_2_); IR *ν*_max_ 3467, 2927, 1780, 1739, 1368, 1250, 1223, 1041 cm^−1; 1^H NMR data (400 MHz, pyridine-*d*_5_), see Table 1; ^13^C NMR data (100 MHz, pyridine-*d*_5_), see Table 2; ESIMS *m/z* 607 [M]^+^; HRESIMS *m/z* 607.1925 [M + Na]^+^ (calcd for C_28_H_37_ClO_11_Na, 607.1922), 609.1892 [M + Na + 2]^+^.

### 3.4. Human Neutrophils Superoxide Generation and Elastase Release

Human neutrophils were obtained by means of dextran sedimentation and Ficoll centrifugation. The assay of O_2_ ^•−^ generation was based on the SOD-inhibitable reduction of ferricytochrome *c*. Degranulation of azurophilic granules was determined by elastase release as described previously [16]. The elastase release experiments were performed using MeO-Suc-Ala-Ala-Pro-Val-*p*-nitroanilide as the elastase substrate. The fMet-Leu-Phe (fMLP), activated by Cytochalasin B (CB), was used as a stimulant. Genistein was used as a standard compound.

## 4. Conclusion

Chemical investigation of the Taiwanese gorgonian *Junceella fragilis* has resulted in the isolation of four new briarane diterpenoids, frajunolides L–O (**1**–**4**). Among them, compounds **1**, **3** and **4** exhibited mild or selective anti-inflammatory activity.

## Supplementary Data

Supplementary data associated with this article can be found in the online version.

## Supporting Information



## Figures and Tables

**Chart 1:**
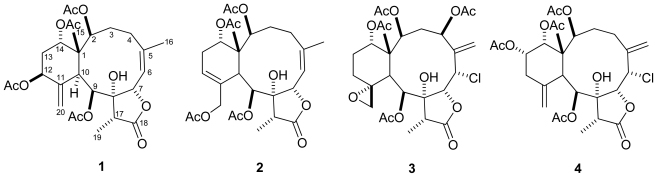
Structures of Frajunolides L–O (**1**–**4**).

**Table 1:** NMR spectroscopic data for compounds **1**–**4**.

	**1**^b^		**2**^b^		**3**^c^		**4**^c^	

Position	δ_H_ (*J* in Hz) a	δ_H_, mult. d	δ_H_ (*J* in Hz)	δ_H_, mult.	δ_H_ (*J* in Hz)	δ_H_, mult.	δ_H_ (*J* in Hz)	δ_H_, mult.
1		47.0, C		45.7, C		48.9, C		48.3, C
2	4.94, t (3.3)	74.2, CH	5.01, m	75.0, CH	6.68, d (8.5)	73.2, CH	6.62, d (8.0)	74.8, CH
3	2.16, m	30.8, CH_2_	2.54, m	33.3, CH_2_	3.58, dd (16.0, 10.5)	37.3, CH_2_	2.88, m	29.3, CH_2_
	1.72, m		1.61, m		2.03, dd (16.0, 8.5)		1.70, m	
4	2.58, m	29.0, CH_2_	1.95, m	29.7, CH_2_	5.90, d (10.5)	77.6, CH	2.52, m	33.4, CH_2_
	2.08, m							
5		145.2, C		146.1, C		144.0, C		144.9, C
6	5.58, d (9.6)	120.0, CH	5.41, d (9.2)	117.7, CH	5.42, d (3.5)	54.2, CH	5.21, d (3.2)	56.2, CH
7	5.28, d (9.6)	78.0, CH	5.32, d (9.2)	79.1, CH	4.94, d (3.5)	85.3, CH	4.92, m	84.9, CH
8		82.6, C		82.5, C		82.7, C		82.1, C
9	5.62, d (5.1)	72.6, CH	5.74, s	71.4, CH	6.30, s	72.6, CH	6.28, s	79.3, CH
10	3.57, d (5.1)	40.6, CH	3.07, s	39.9, CH	3.62, s	42.4, CH	3.82, s	44.6, CH
11		146.3, C		134.2, C		58.2, C		147.0, C
12	5.32, m	71.5, CH	5.85 br, s	127.6, CH	2.27, m	31.6, CH_2_	2.63, t (12.4)	38.6, CH_2_
					1.28, m		2.49, m	
13	2.20, m	33.5, CH_2_	2.28, m	28.1, CH_2_	1.94, m	25.5, CH_2_	5.27, ddd (3.2, 5.2, 12.0)	70.1, CH
	1.95, m		2.11, m					
14	4.77 br, s	73.6, CH	4.75 br, s	73.7, CH	5.25, s	75.2, CH	5.66, s	73.8, CH
15	1.15, s	15.4, CH_3_	0.99, s	16.2, CH_3_	1.31, s	15.2, CH_3_	1.27, s	14.4, CH_3_
16	2.03, s	27.0, CH_3_	1.99, s	29.0, CH_3_	5.83, s	125.5, CH_2_	4.92, s	118.3, CH_2_
					5.42, s		5.49, s	
17	2.54, q (6.9)	43.2, CH	2.45, q (7.2)	44.7, CH	3.44, q (7.0)	51.8, CH	3.41, q (7.6)	51.4, CH
18		175.9, C		174.7, C		176.4, C		175.8, C
19	1.15, d (6.9)	6.7, CH_3_	1.17, d (6.8)	8.7, CH_3_	1.41, d (7.0)	7.3, CH_3_	1.26, d (7.6)	6.7, CH_3_
20	5.34, s	118.3, CH_2_	4.67, d (12.0)	68.7, CH_2_	2.84, d (4.0)	52.6, CH_2_	4.92, s	113.1, C
	5.30, s		5.02, d (12.0)		2.59 br, s		5.19, s	
OAc	2.21, s	170.4, C	2.16, s	169.9, C	2.30, s	172.5, C	2.28, s	171.8, C
	2.13, s	170.4, C	2.07, s	169.7, C	2.30, s	171.1, C	2.09, s	170.9, C
	1.98, s	170.3, C	2.01, s	169.3, C	2.11, s	171.1, C	2.07, s	170.8, C
	1.94, s	168.9, C	1.92, s	168.4, C	1.99, s	170.3, C	1.99, s	170.3, C
		21.7, CH_3_		23.1, CH_3_		22.2, CH_3_		21.9, CH_3_
		21.2, CH_3_		22.9, CH_3_		21.9, CH_3_		21.1, CH_3_
		21.2, CH_3_		22.8, CH_3_		21.8, CH_3_		21.0, CH_3_
		21.1, CH_3_		22.7, CH_3_		21.5, CH_3_		20.9, CH_3_
8-OH							8.05 br, s	

a Data were recorded at 400 and/or 500 MHz in CDCl_3_;

b In CDCl_3_;

c In pyridine-*d*_5_;

d Data recorded at 100 and/or 125 MHz and were assigned by DEPT, COSY, HSQC, and HMBC experiments.

**Table 2:** Effects of compounds on superoxide anion generation and elastase release by human neutrophils in response to fMet-Leu-Phe (fMLP)/Cytochalasin B (CB).

Compounds	Superoxide anion Inh % a	Elastase release Inh % a
**1**	18.7 ± 2.6 **	16.2 ± 0.7 ***
**2**	2.0 ± 2.3	13.3 ± 3.1 *
**3**	0.6 ± 1.5	22.3 ± 7.7
**4**	8.3 ± 3.6	17.2 ± 6.7 *
Genistein	65.0 ± 5.7	51.6 ± 5.9

a Percentage of inhibition Inh % at 10 μg/mL concentration. Results are presented as mean ± S.E.M. (*n* = 3).

* *P* < 0.05,

** *P* < 0.01,

*** *P* < 0.001 compared with the control value.
