# Supplementary material for: Frajunolides L–O, Four New 8-Hydroxybriarane Diterpenoids from the Gorgonian Junceella fragilis
Source: Mar Drugs. 2011 Sep 2;9(9):1477–86. doi: 10.3390/md9091477 (PMC3225929; doi:10.3390/md9091477)

Supporting Information

Frajunolides L-O, Four New 8-Hydroxybriarane Diterpenoids from the Gorgonian *Junceella fragilis*

Chia-Ching Liaw[1,2], Yao-Haur Kuo[3], Yun-Sheng Lin[2], Tsong-Long Hwang[4], and Ya-Ching Shen*[,1]

[1] School of Pharmacy, College of Medicine, National Taiwan University, Taipei 100, Taiwan, Republic of China; E-Mail: ycshen@ntu.edu.tw (Y.-C.S.); biogodas@hotmail.com (C.-C.L.)

[2] Department of Marine Biotechnology and Resources, National Sun Yat-Sen University, Kaohsiung 804, Taiwan, Republic of China; E-Mail: x00010106@meiho.edu.tw (Y.-S.L.)

[3] National Research Institute of Chinese Medicine, Taipei 112, Taiwan, Republic of China; E-Mail: kuoyh@nricm.edu.tw (Y.-H.K.)

[4] Graduate Institute of Natural Products, Chang Gung University, Taoyuan 333, Taiwan, Republic of China; E-Mail: htl@mail.cgu.edu.tw (T.-L.H.)

*Author to whom correspondence should be addressed; Tel.: +886-2-23123456, ext. 62226; Fax: +886-2-2391-9098. (School of Pharmacy, College of Medicine, National Taiwan University, Jen-Ai Rd. Sec. 1, Taipei 100, Taiwan, Republic of China)

List of Supporting Information

Figure S1. ^{1}H NMR spectrum (400 MHz, CDCl3) of frajunolide L (**1**)

Figure S2. ^{13}C NMR spectrum (100 MHz, CDCl3) of frajunolide L (**1**)

Figure S3. ^{1}H NMR spectrum (400 MHz, CDCl3) of frajunolide M (**2**)

Figure S4. ^{13}C NMR spectrum (100 MHz, CDCl3) of frajunolide M (**2**)

Figure S5. ^{1}H NMR spectrum (400 MHz, pyridine-d_5) of frajunolide N (**3**)

Figure S6. ^{13}C NMR spectrum (100 MHz, pyridine-d_5) of frajunolide N (**3**)

Figure S7. ^{1}H NMR spectrum (400 MHz, pyridine-d_5) of frajunolide O (**4**)

Figure S8. ^{13}C NMR spectrum (100 MHz, pyridine-d_5) of frajunolide O (**4**)

Figure S1. ^{1}H NMR (400 MHz, $CDCl_3$) of frajunolide L (**1**)

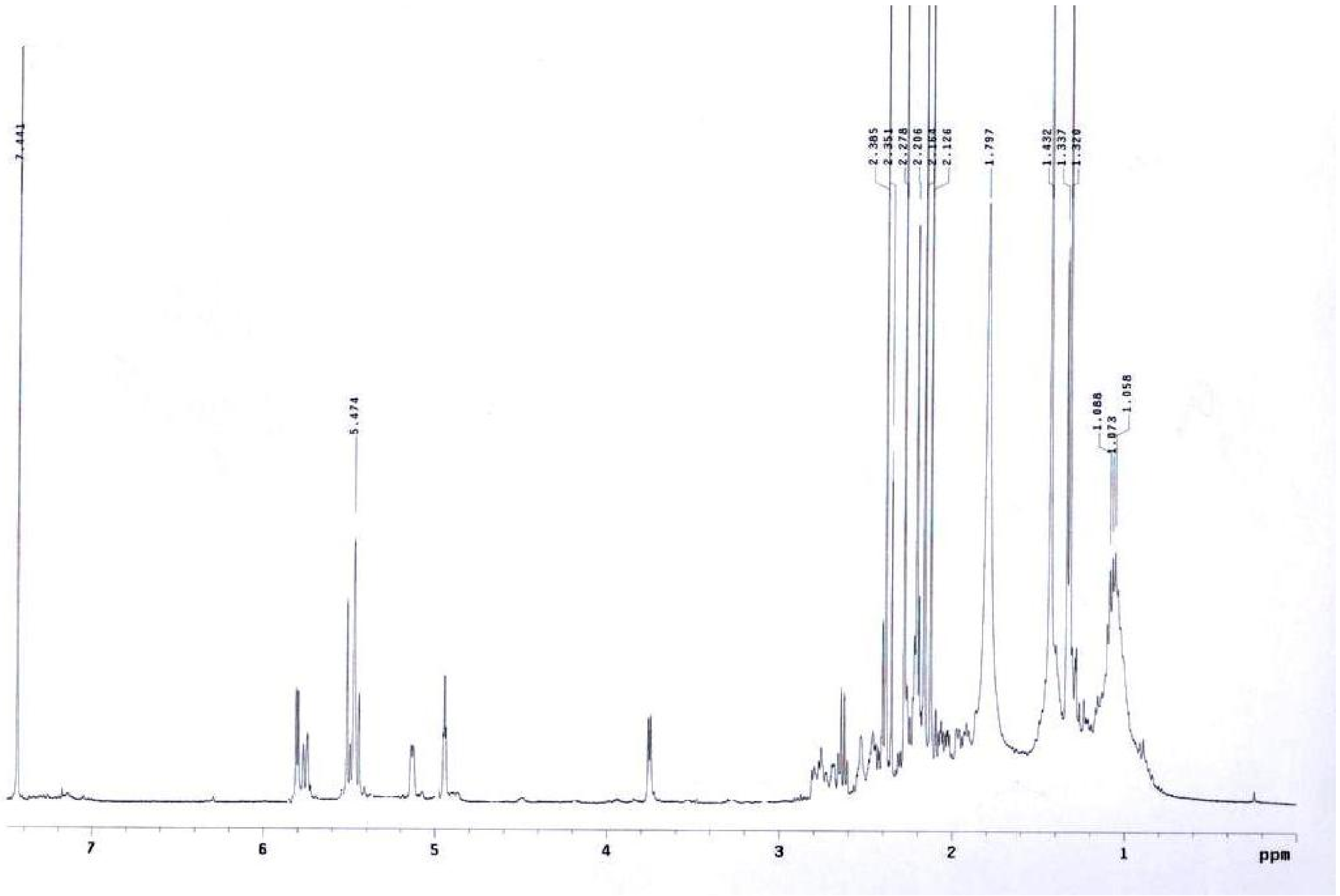

Figure S2. ^{13}C NMR (100 MHz, $CDCl_3$) of frajunolide L (**1**)

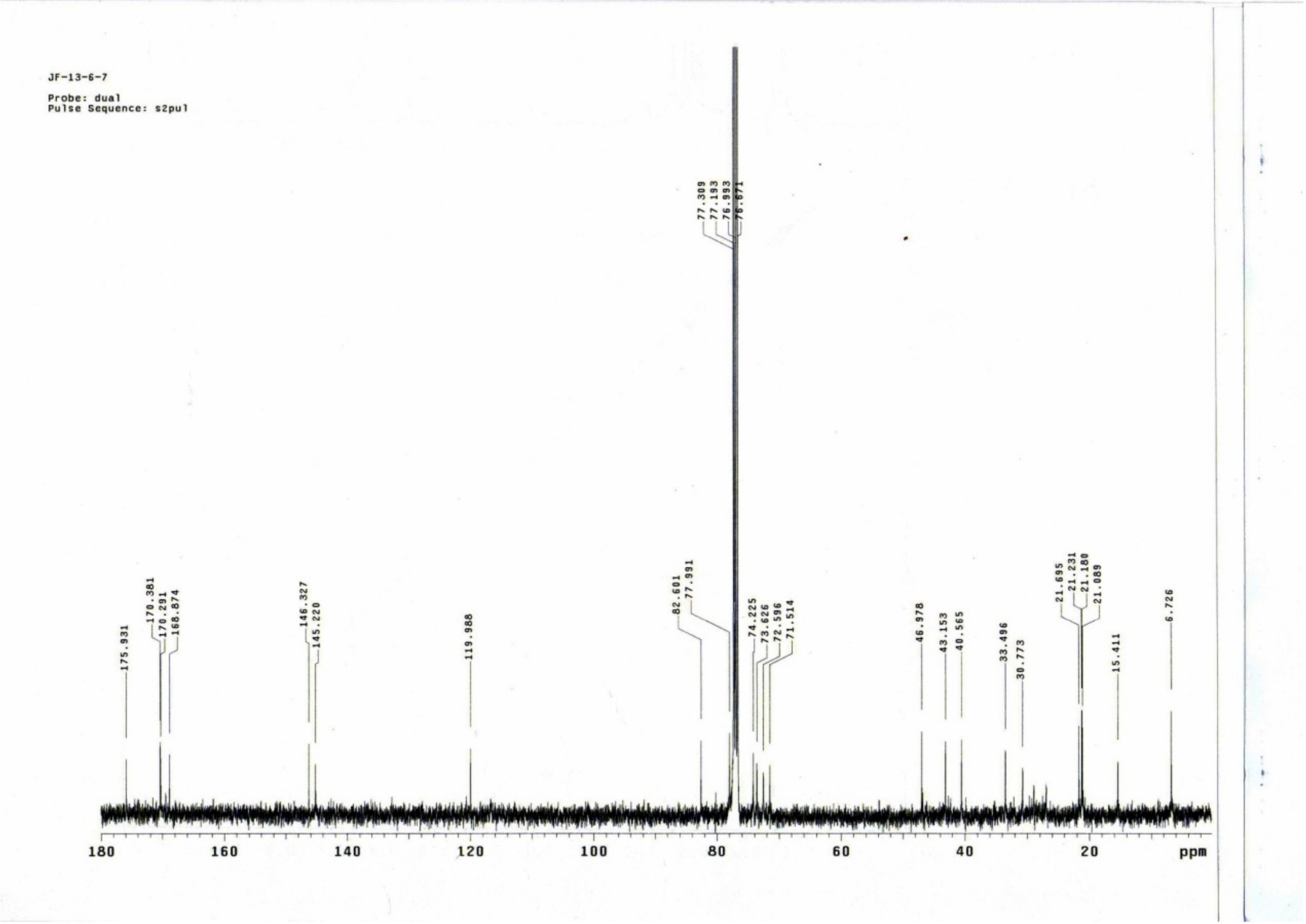

Figure S3. ^{1}H NMR (400 MHz, $CDCl_3$) of frajunolide M (**2**)

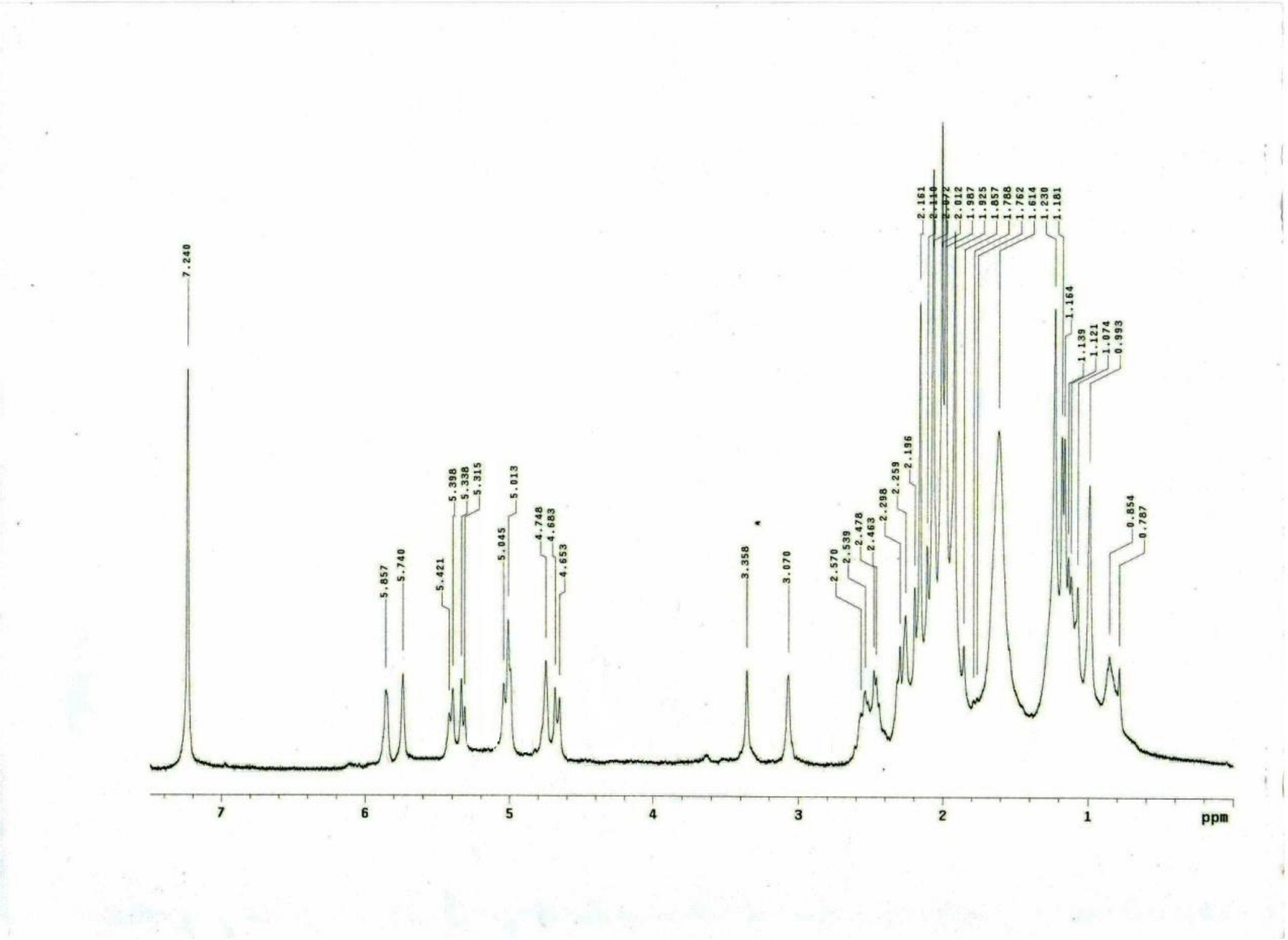

Figure S4. ^{13}C NMR (100 MHz, $CDCl_3$) of frajunolide M (**2**)

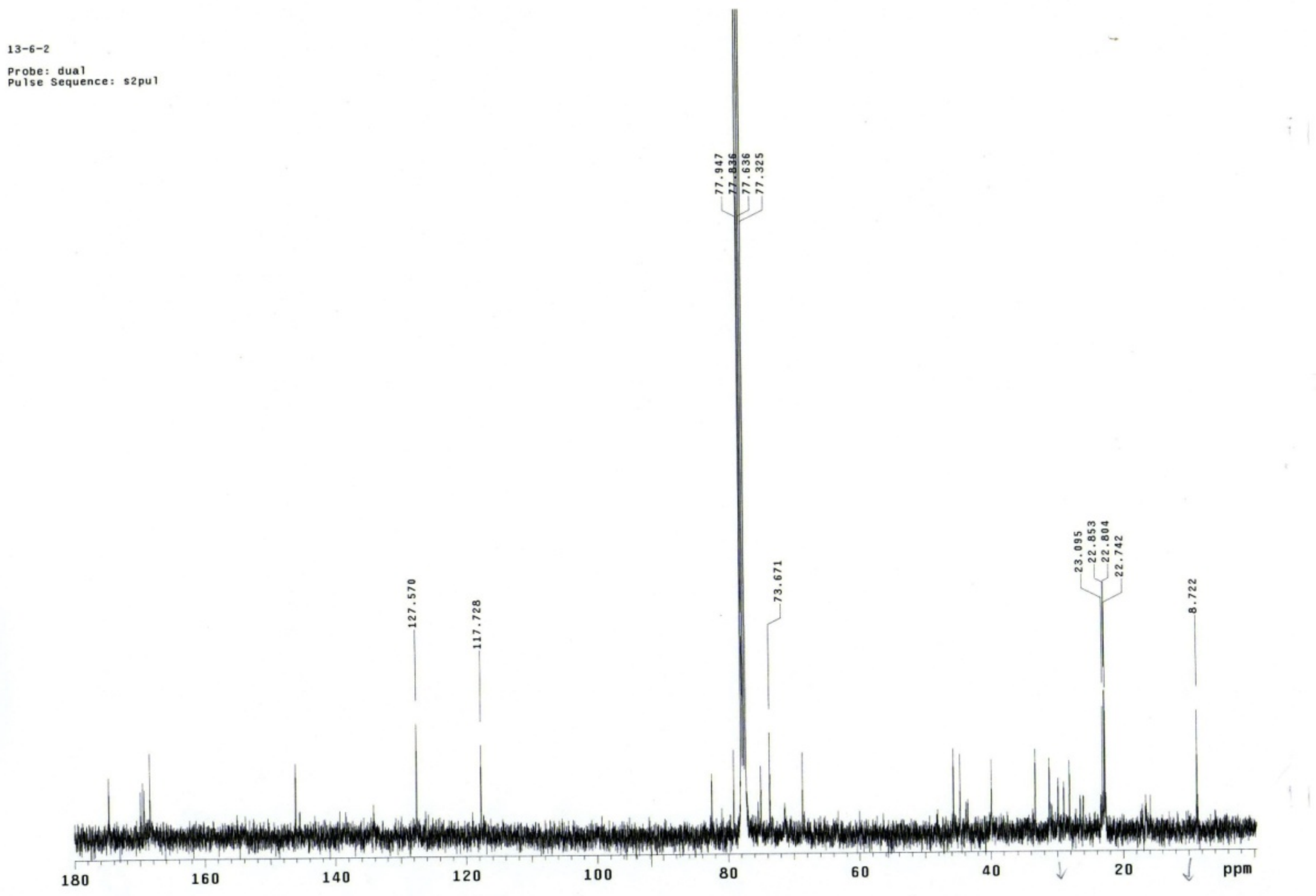

Figure S5. ^{1}H NMR (400 MHz, pyridine-d_5) of frajunolide N (**3**)

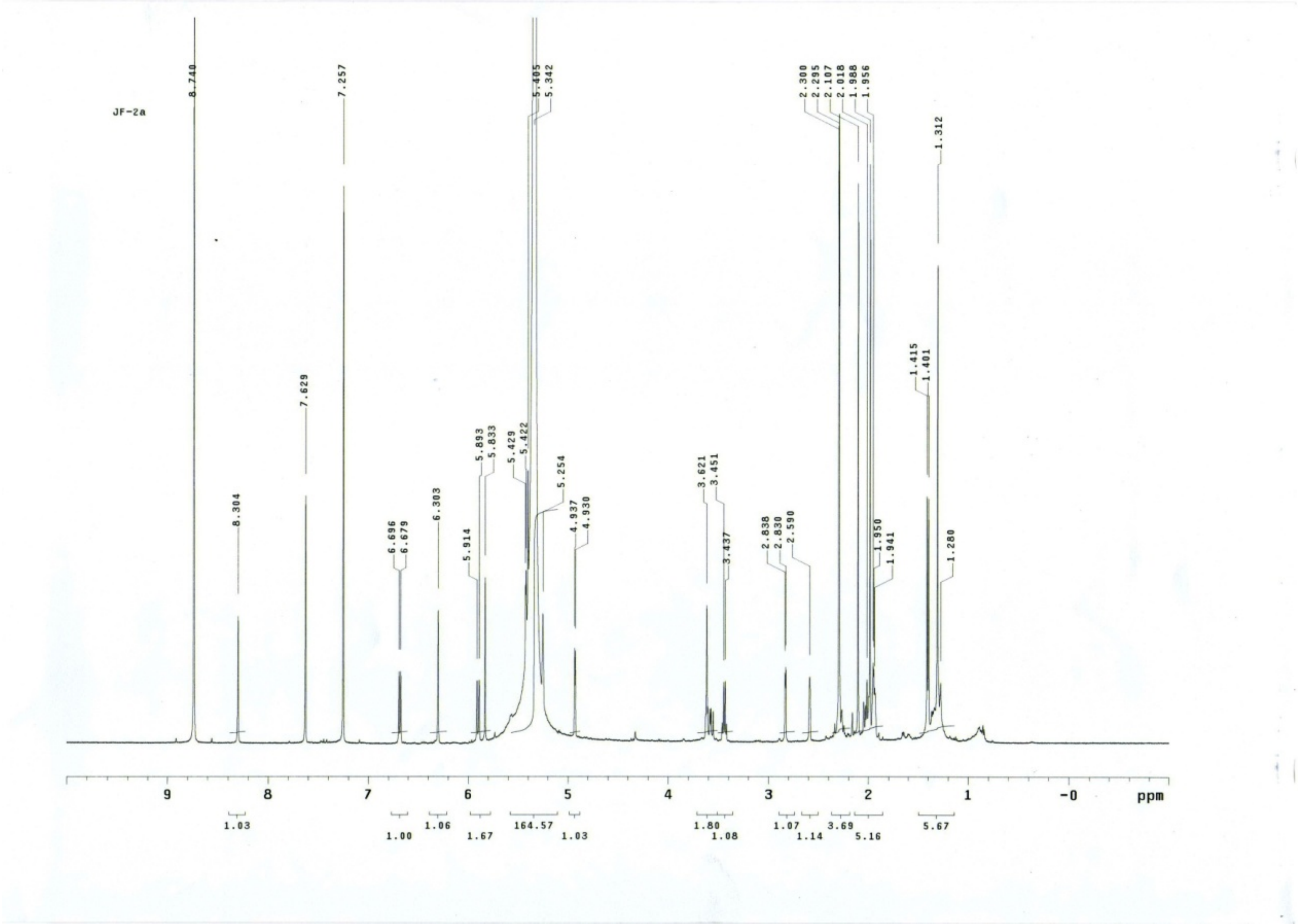

Figure S6. ^{13}C NMR (300 MHz, pyridine-d_5) of C frajunolide N (**3**)

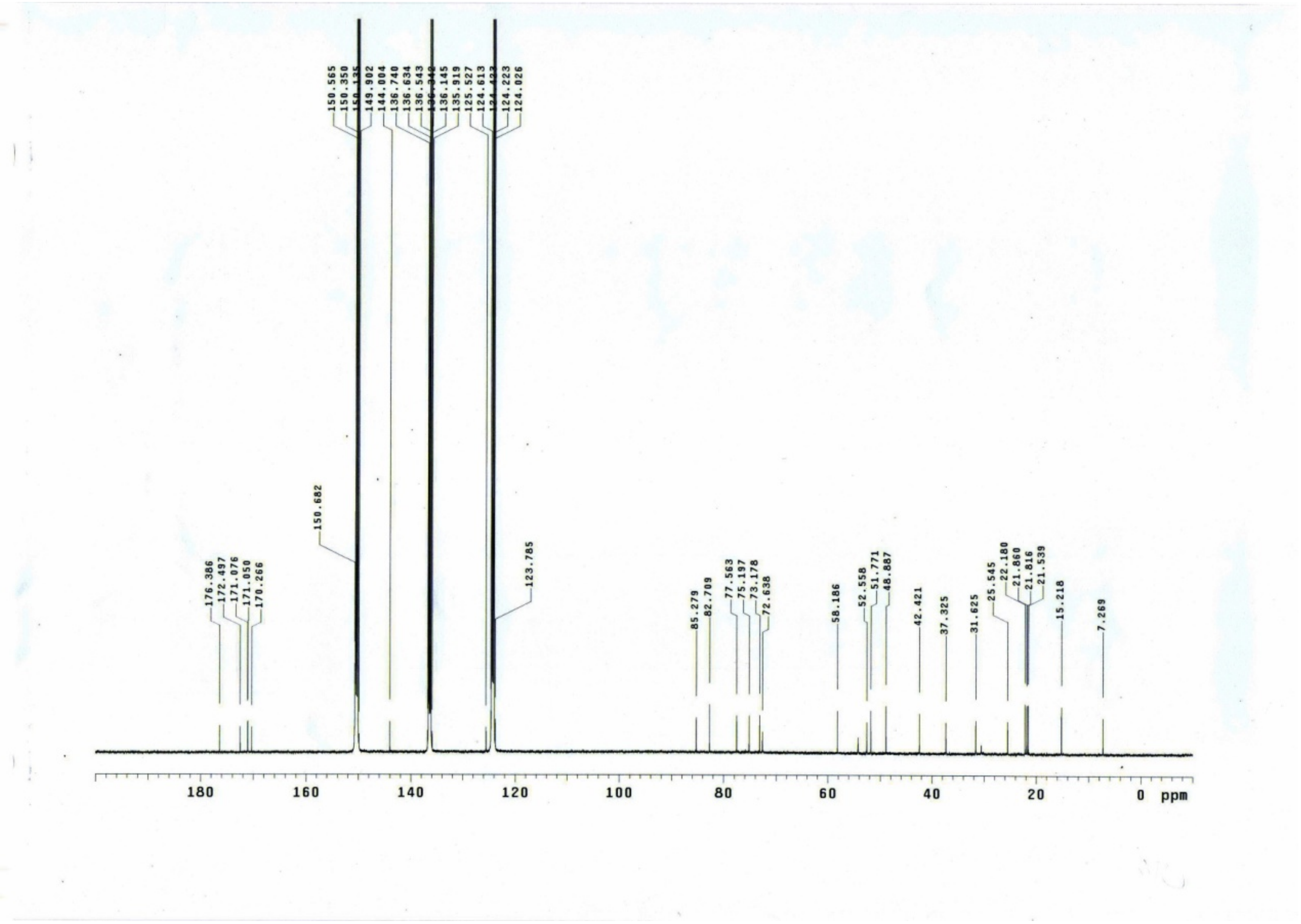

Figure S7. ^{1}H NMR (400 MHz, pyridine-d_5) of frajunolide O (**4**)

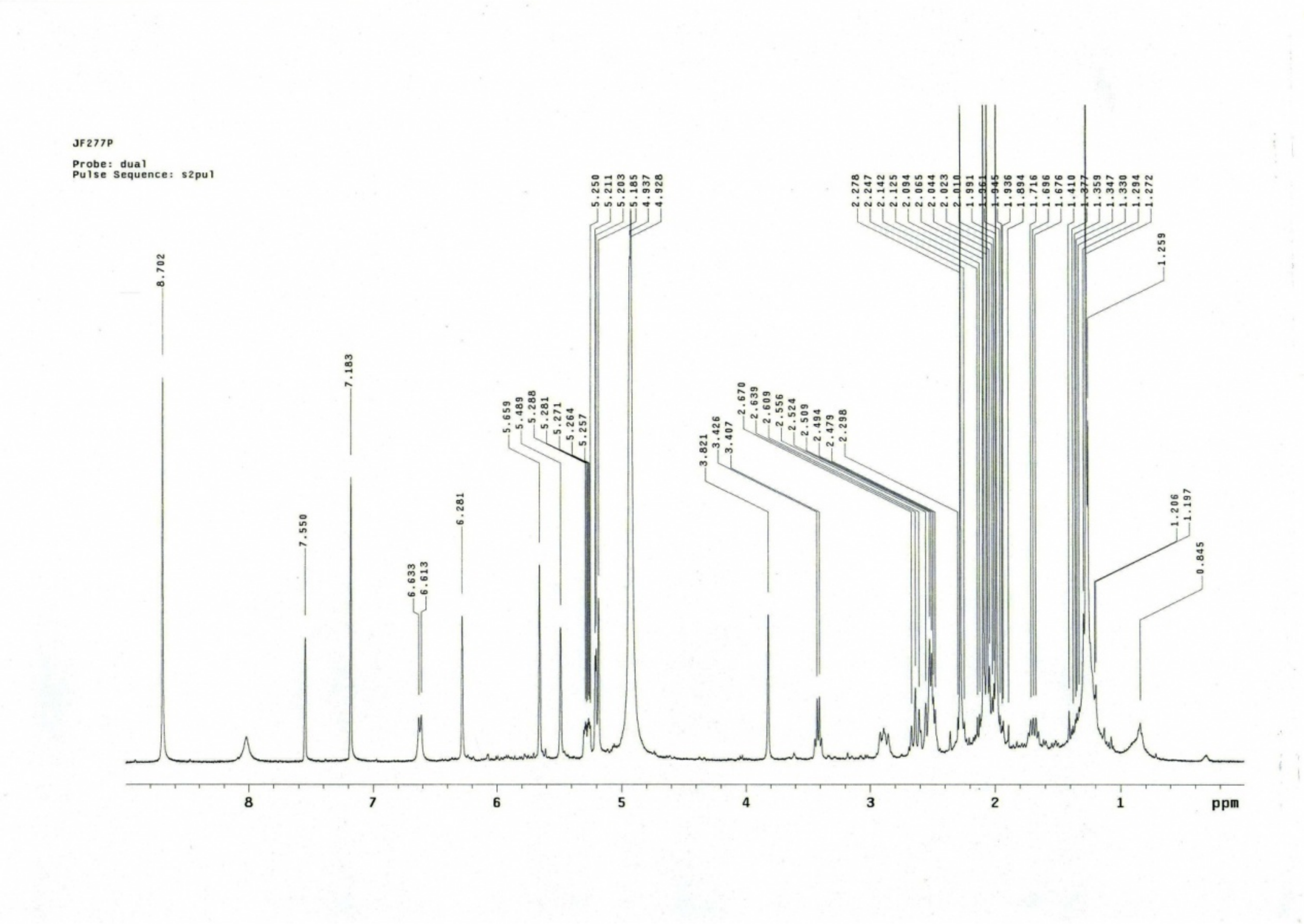

Figure S8. ^{13}C NMR (300 MHz, pyridine-d_5) of frajunolide O (**4**)

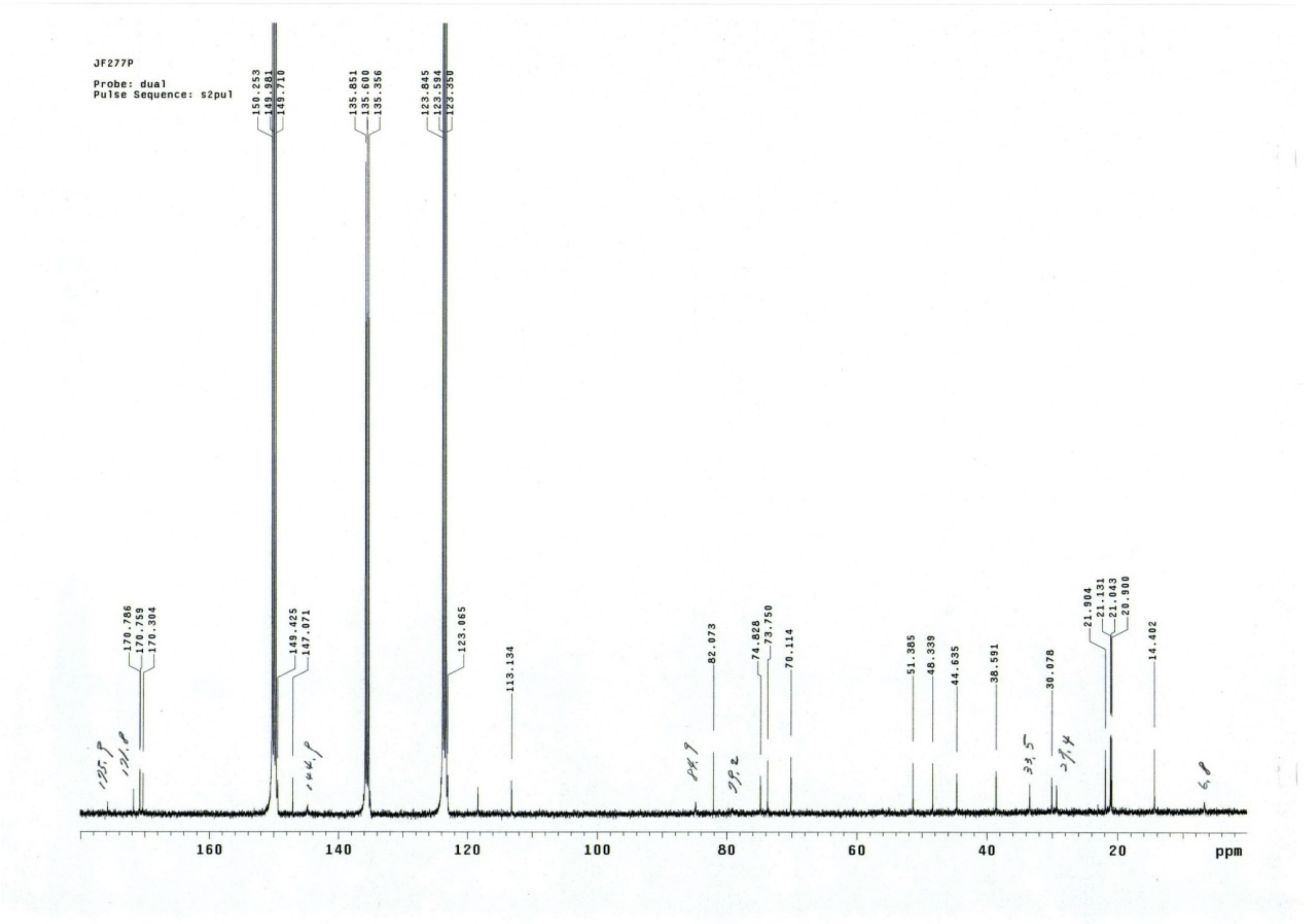

Supplement: Supplementary file 1 [file marinedrugs-09-01477-s001.pdf]
